# The safety and efficacy of acupuncture for erectile dysfunction: A network meta-analysis

**DOI:** 10.1097/MD.0000000000014089

**Published:** 2019-01-11

**Authors:** Jisheng Wang, Yu Zhou, Hengheng Dai, Binghao Bao, Jin Dang, Xiao Li, Bin Wang, Haisong Li

**Affiliations:** a Graduate School of Beijing University of Chinese Medicine; b Department of Andrology, Dongzhimen Hospital, Beijing University of Chinese Medicine; c Department of Dermatology, Beijing Changping Hospital of Traditional Chinese Medicine, Beijing, China.

**Keywords:** acupuncture, erectile dysfunction, network meta-analysis, protocol

## Abstract

**Background:**

Erectile dysfunction is a common male disease, the constant pace of life, increasing pressure on life, and changes in diet, living environment, lifestyle, etc., lead to an increase in the number of patients with erectile dysfunction (ED). Acupuncture has been widely used in clinical trials of ED in recent years. There are many clinical trials that confirm that acupuncture can improve male erectile function. This study used a network meta-analysis (NMA) to compare the effectiveness and safety of different forms of acupuncture on ED.

**Methods:**

We will search for PubMed, Cochrane Library, AMED, EMBASE, WorldSciNet; Nature, Science online and China Journal Full-text Database (China National Knowledge Infrastructure), China Biomedical Literature CD-ROM Database (CBM), and related randomized controlled trials (RCTs) included in the China Resources Database. The time is limited from the construction of the library to December 2018. The quality of the included RCTs will be evaluated with the risk of bias tool and evidence will be evaluated by Grading of Recommendations Assessment, Development and Evaluation. STATA 13.0 and WinBUGS 1.4.3 through the GeMTC package will be used to perform an NMA to synthesize direct and indirect evidence.

## 1 Introduction

Erectile dysfunction (ED) is a disease that, with normal sexual stimulation, the penis cannot achieve or maintain a sufficient erection, which make the patients cannot perform normal sexual intercourse.^[1]^ ED is now a common male disease, which, because of the accelerating pace of life, increasing pressure and changes in diet, living environment, lifestyle, etc., has an increasing morbidity. According to the statistics, there are about 150 million men worldwide suffering from the disease, which make up 20% of global adult males, and the statistical data are increasing year after year.^[2,3]^ The total prevalence of ED in Chinese men is about 26.1%, in addition, the prevalence of men over 40 years old is as high as 40.2%.^[4]^ The pathogenesis of the disease is related to multiple factors, the main causes of which are psychological factors, organic factors, and psycho-organic mixing factors.^[5,6]^ Among them, psychological factors are mainly caused by the emotion, mental, society, losing control of sexual behavior, and the lack of sexual knowledge.^[7]^ And the organic factors are mainly related to kinds of diseases, drugs, trauma, and surgery. ED not only impacts the quality of life of the male patients, the harmony of family and society, but also causes the pain of their body and mind. At present, with the rapid development of current material and spiritual civilization, concern of the prevention and treatment of ED has been increasing.^[8,9]^

There are lots of therapies of this disease, which are mainly divided into noninvasive ways and invasive ways.^[10]^ Noninvasive therapy is mainly based on oral type 5 phosphodiesterase inhibitor (PDE5-Is) drugs as the main treatment, which has obvious short-term efficacy; however, the long-term efficacy does not respond well.^[11]^ Besides, this kind of therapy is prone to headaches, myalgia, and other side-effects of pain. It has recently been reported that PDE5-Is may be associated with hearing impairment and may increase the risk of severe hypotension.^[12]^ Invasive therapies mainly include intracavernosal injection of vasoactive drugs, vacuum suction devices, and penile prosthesis implantation. These invasive therapies could bring pain and many adverse reactions to patients.^[13,14]^ Although there are many methods currently used for ED treatment, the efficacy of these therapies is not stable, the symptoms are prone to recurrence, and many patients feel intolerable.

ED belongs to the category of impotence and “jinwei” in traditional Chinese medicine (TCM). TCM has a long history of understanding and treatment of this disease, and has accumulated rich experience, which has been highly affirmed on the efficacy.^[15]^ Acupuncture, an essential part of TCM, has been widely used in clinical trials of ED in recent years.^[16]^ Vast studies have shown that acupuncture Tianshu (ST25), Zusanli (ST36), and Taichong (LR3) can regulate neurotransmitter 5-HT levels and reduce neurological sensitivity, which could extend the ejaculation latency to some extent. Besides, studies also have shown that acupuncture middle pole (RN3) and Guanyuan (RN4) can improve the blood flow supply of the corpus cavernosum, which could shorten the time that the penis reaches the erection and increase the hardness of the penis erection.^[17,18]^

After a preliminary search and analysis of database resources, we found that randomized controlled trials (RCTs) of acupuncture for ED are gradually increasing.^[19,20]^ However, most clinical trials confront with the inferior quality of the studies with small sample size and the insufficiency of evidence-based exploration because of the limitation of the size and number of clinical centers. Therefore, we expect to use the network meta-analysis (NMA) to evaluate the efficacy and safety of acupuncture in the treatment of ED, and provide a basis for clinical application.

## 2 Methods

This is a systematic review and ethical approval was not necessary.

### 2.1 Study registration

This systematic review protocol has been registered on PROSPERO as CRD42018092783 (https://www.crd.york.ac.uk/PROSPERO/display_record.php?RecordID=111738).

### 2.2 Eligibility criteria

#### 2.2.1 Type of study

RCTs of acupuncture (electroacupuncture, fire needle, plum blossom needle, acupuncture, embedding) or acupuncture combined with other effective interventions (drugs or other) as treatment methods, including the control group (effective methods other than acupuncture). The language is limited to Chinese and English. Non-RCTs, quasi-RCTs, case series, case reports, and crossover studies will be excluded.

#### 2.2.2 Participants

The patient must be 18 years of age or older, with no restrictions on the stage or severity of the disease, no matter if there is a traditional disease. The ED must be diagnosed according to at least one internationally or nationally authorized diagnostic criteria. International erectile function score (International Index of Erectile Function 5, IIEF-5) <21. The group was well balanced when enrolled.

#### 2.2.3 Types of interventions

##### 2.2.3.1 Experimental interventions

The acupuncture treatment of ED will be regarded as experimental interventions, which includes the use of acupuncture, electroacupuncture, fire acupuncture, plum needle, the massage on the related acupoints. Besides, acupuncture combined with other effective interventions to treat ED will also be included. Considering that the theory of pharmaco-acupuncture and point injection belongs to another part of TCM, so they will be considered for exclusion.

##### 2.2.3.2 Control interventions

As for the control interventions, who accepted virtual acupuncture treatment can be used as a placebo-controlled or did not get any treatment or other conventional treatments as a blank control would be adopted. However, once they had accepted acupuncture combined medication or other therapy of TCM, the trials will be rejected.

The following treatment comparisons will be investigated:

(1) acupuncture versus no treatment;
(2) acupuncture versus placebo/sham acupuncture;
(3) acupuncture versus drug therapy;
(4) acupuncture versus other active therapies; and
(5) acupuncture with another active therapy versus the same therapy alone.

#### 2.2.4 Outcomes

The primary outcome is based on the International Emotional Function Score (IIEF-5) Efficacy Evaluation Criteria. Healing: IIEF-5 score after treatment is ≥22 points; markedly effective: IIEF-5 points <22 points after treatment, score improvement ≥60%; effective: IIEF-5 points after treatment <22 points, points improved <60%, but ≥30%; invalid: after treatment, IIEF-5 points <22 points, points improved <30%.

The second outcome is based on TCM syndrome evaluation criteria. Healing: The clinical symptoms and signs of TCM disappear or disappear, and the syndrome score is reduced by ≥90%; significant effect: the clinical symptoms and signs of TCM are obviously improved, and the syndrome score is reduced by ≥60%; effective: Chinese medicine clinical symptoms and signs have improved, syndrome scores decreased by <60%, but ≥30%; invalid: the clinical symptoms and signs of TCM were not improved, even worse, and the syndrome score was reduced by <30%. Integral variation formula (Nimodipine method: [(pretreatment score − post-treatment score)/pretreatment score] × 100%).

#### 2.2.5 Data source

Database Search: PubMed, Cochrane Library, AMED, EMBASE, WorldSciNet, Nature, Science online and China Journal Full-text Database (China National Knowledge Infrastructure), China Biomedical Studies CD-ROM Database (CBM), China Resources Database. A study review of clinical studies on acupuncture (or acupuncture) for the treatment of ED published in domestic and foreign biomedical journals from the establishment of the library to December 2018. Based on the standards of the Cochrane Collaboration Workbook of the International Evidence-Based Medicine Center, a manual and computer-based approach is used to conduct relevant studies’ searches. Search terms include: acupuncture, electroacupuncture, fire needle, plum blossom needle, skin needle, sexual dysfunction, erectile dysfunction, impotence, and jingwei. Manually search for the titles and abstracts of the relevant researches in “Chinese Men's Science Magazine,” “Chinese Men's Science Journal,” “Chinese Acupuncture and Moxibustion,” and “Acupuncture Research.”

#### 2.2.6 Study selection

Applying the EndnoteX7 software to manage the included references. Two qualified evaluators independently screened the titles and abstracts of the selected studies, excluding duplicates and documents that did not significantly conform to the study. After a preliminary evaluation, the selected documents will be read one by one. Exclusions were based on inclusion criteria for uncontrolled studies, no randomization, inconsistent assessment criteria, and similar data. If there are different opinions, the third reviewer should be consulted. Studies information and data extraction were carried out on the final included studies, including the experimental methods of the study, the basic information of the included cases, the observation period, the intervention methods, observation indicators, and test results of the treatment group and the control group.

#### 2.2.7 Risk of bias

The quality of the studies will be assessed by using the Cochrane Handbook 5.1.0.^[21]^ The assessment will include random sequence generation, randomization correctness, allocation scheme hiding, blinding of patients and implementers, accuracy of data results, and other risk of bias. The risk of low bias is expressed as “low risk” and the risk of high bias is expressed as “high risk.” The information provided in the studies is inaccurate or does not provide sufficient information for the bias assessment to be expressed as “unclear risk.” The above content evaluation was independently evaluated by 2 researchers, and any differences will be resolved through discussions with the third reviewer.

#### 2.2.8 Statistical analysis

##### 2.2.8.1 Pair-wise meta-analysis

The numerical variable will be expressed as the standardized mean difference (SMD) with a 95% confidence interval (CI). The heterogeneity of each pair-wise comparison will be tested by chi-squared test (test level α = 0.1). If there is no heterogeneity, a fixed-effect model will be used. If there is significant heterogeneity between a group of studies, we will explore the reasons for the existence of heterogeneity from various aspects such as the characteristics of the subjects and the degree of variation of the interventions. Sensitivity analysis or meta-regression and subgroup analysis to explore possible sources of heterogeneity if it is necessary. We will use qualitative analysis of the funnel plot and graph symmetry to assess publication bias. Quantitative methods such as Begg testing and Egger testing will be used to help assess publication bias in the application.

##### 2.2.8.2 Network meta-analysis

We will use GeMTC 0.14.3 software to analyze the data. STATA 13.0 and WinBUGS 1.4.3 will be used to perform NMA to synthesize direct and indirect evidence. The NMA will mainly use the Bayesian Markov chain–Markov Chain Monte Carlo random-effect model and simulate with 5 chains.^[22]^ The convergence of the simulation will be evaluated using the potential reduction factor and the Gelman-ubin-rooks diagram. The choice of the final model will depend on deviance information criterion (DIC) value. In general, models with smaller DIC values are better. The total effective rate is counted, and the odds ratio is used to analyze the statistic. The effect size is expressed in 95% CI, and the numerical variable is expressed as SMD. The treatment level for each result will operate on the Cumulative Sorting Curve (SUCRA) interface. The evidence relationship incorporated into the study will be calculated by STATA. If there is a “closed loop,” the node splitting method will be used to evaluate the inconsistency of each loop.

##### 2.2.8.3 Quality of evidence

The Grading of Recommendations Assessment, Development and Evaluation (GRADE) method will also be used to assess the quality of evidence for key outcomes. This assessment will be conducted through a Guideline Development Tool (GRADEpro GDT, https://gradepro.org/).

## 3 Discussion

Erectile is a very complicated physiological process; thus, the cause of ED is also very complicated, which involves numerous aspects including psychological factors, vascular factors, endocrine factors, and neurological factors. Any abnormality in one of these aspects can cause disorders in male erectile function.^[23,24]^ In this regard, TCM believes that the etiology and pathogenesis of ED is related to spleen and kidney deficiency and qi and blood block.^[25]^ Acupuncture at the corresponding acupoints can play the role of strengthening the spleen and kidney, promoting blood circulation and collaterals, and at the same time improve the mood and achieve the purpose of treatment.^[26]^ Judging from the view of modern medicine, acupuncture treatment can regulate nerve sensitivity and improve the blood supply of peripheral blood vessels, which achieves the efficacy.^[27]^ Recent studies have shown that acupuncture can improve male erectile function to a some extent, and related experimental studies are constantly increasing.^[18]^

Although many studies have evaluated the effectiveness of acupuncture in the treatment of ED, there is still a lack of evaluation and comparison of various treatments. To the best of our knowledge, NMA has not been used in recent years to compare the effectiveness of acupuncture in the treatment of ED. The results of NMA can provide a possible ranking for acupuncture treatment of ED. Besides, we will use the GRADE method to assess the quality of evidence for the key outcomes. We hope that the results will provide clinicians with the best choice for treatment of ED and provide research directions. Although we will conduct a comprehensive search in this study, languages other than Chinese and English will be limited, which will lead to some bias. In addition, the studies on acupuncture treatment of ED have the deficiency of small sample size and low overall quality, which may affect the authenticity of this study. Therefore, we hope that in the future, there will be more rigorous and reasonable multicenter RCTs to explore the clinical efficacy of acupuncture for ED, and make the conclusion more objective and reasonable.

## Author contributions

**Data curation:** Jisheng Wang, Yu Zhou.

**Formal analysis:** Jin Dang, Xiao Li.

**Funding acquisition:** Haisong Li.

**Project administration:** Bin Wang.

**Supervision:** Haisong Li, Bin Wang

**Validation:** Haisong Li.

**Writing – original draft:** Jisheng Wang, Yu Zhou.

**Writing – review & editing:** Hengheng Dai, Binghao Bao.
